# Relationship between Work Hours and Smoking Behaviors in Korean Male Wage Workers

**DOI:** 10.1186/2052-4374-25-35

**Published:** 2013-11-19

**Authors:** Sung-Mi Jang, Eun-hee Ha, Hyesook Park, Eunjeong Kim, Kyunghee Jung-Choi

**Affiliations:** 1 Department of Preventive Medicine, Ewha Womans University School of Medicine, Seoul, South Korea

**Keywords:** Work hours, Working hours, Smoking, Precarious employment, Job satisfaction

## Abstract

**Objectives:**

The purposes of this study are 1) to measure the prevalence of smoking according to weekly work hours by using data from the Korean Labor and Income Panel Study (KLIPS), and 2) to explain the cause of high smoking prevalence among those with short or long work hours by relative explanatory fraction.

**Methods:**

Data from a total of 2,044 male subjects who responded to the questionnaire in the 10th year (2007) and 11th year (2008) of the Korean Labor and Income Panel Study were used for analysis. Current smoking, smoking cessation, continuous smoking, start of smoking, weekly work hours, occupational characteristics, sociodemographic and work-related factors, and health behavior-related variables were analyzed. Log-binomial regression analysis was used to study the relationship between weekly work hours and smoking behaviors in terms of the prevalence ratio.

**Results:**

The 2008 age-adjusted smoking prevalence was 64.9% in the short work hours group, 54.7% in the reference work hours group, and 60.6% in the long work hours group. The smoking prevalence of the short work hours group was 1.39 times higher than that of the reference work hours group (95% confidence interval of 1.17-1.65), and this was explained by demographic variables and occupational characteristics. The smoking prevalence of the long work hours group was 1.11 times higher than that of the reference work hours group when the age was standardized (95% confidence interval of 1.03-1.19). This was explained by demographic variables. No independent effects of short or long work hours were found when the variables were adjusted.

**Conclusion:**

Any intervention program to decrease the smoking prevalence in the short work hours group must take into account employment type, job satisfaction, and work-related factors.

## Introduction

Since the 1970s, interest in the effects of long work hours on health has been growing. This research area became more active when the European Community Directive on Working Time enacted provisions on labor hours in 1993, limiting the work week to a maximum of 48 hours and mandating break time for at least 11 hours a day [1,2]. Many studies have found a relationship between long work hours and cardiovascular disease [3-5]. Long work hours have also been linked to musculoskeletal disease, depression, obesity, decreased labor concentration, increased fatigue, decreased cognitive abilities and judgment, and increased injury [6-10]. On the other hand, some studies have reported that long work hours are not related to cardiovascular disease, type II diabetes, or physical symptoms [11,12].

Smoking causes various cancers, coronary artery disease, and chronic lung disease [13], and is a risk factor that can be controlled. One study measured the disease burden of smoking-related lung cancer as about 96.6 person-years in disability-adjusted life years (DALYs) per 100,000 people in Korea, and about 85.5 person-years in healthy life years (HeaLYs) [14]. Another study that measured the disease burden of smoking-related premature death in Korea reported that 60.9% of premature deaths in males and 17.7% of premature deaths in females could be prevented by quitting smoking [15].

Some studies have investigated the relationship between long work hours and smoking behavior, with inconsistent results. Some studies have reported higher smoking risks in those who work over 40 hours per week than in those who work between 30 and 40 hours [16], and that long work hours are correlated with a lower probability of quitting smoking [17]. Another study found no relationship between long work hours and smoking [18,19]. One review journal reported continued controversy over the relationship between long work hours and smoking [8]. This discordance between study results may come from a lack of well-designed studies [6].

The purposes of this study are 1) to measure the prevalence of smoking according to weekly work hours, and 2) to explain the cause of high smoking prevalence among those with short or long work hours by relative explanatory fraction.

## Materials and methods

### Study subjects

Data from the Korean Labor and Income Panel Study (KLIPS) was used in this study. KLIPS is a longitudinal survey of panel sample members representing 5,000 households in non-rural districts of Korea. The characteristics of each household, economic activity, labor market transfer, earnings and spending, education and occupational training, and social life are studied annually [20]. Among these, the data from the 10th year (2007) and the 11th year (2008) were used for this study.

Among the 11,855 respondents in the 10th year and 11,734 respondents in the 11th year, there were, respectively, 2,790 and 2,658 male workers between the ages of 25 and 64. Among these, 2,323 male workers aged 25 to 64 were continuously studied from the 10th year to the 11th year. Females were excluded because there was a previous study that stated that women's smoking behaviors were not properly reflected in family research [21]. For weekly work hours, working 12 hours a day for 7 days a week adds up to 84 hours per week. Any work hours that exceeded this value were considered extreme and were thus excluded from analysis. Also excluded were those with workers hired after the year 2007, and professional soldiers. As a result, a total of 2,044 male workers were analyzed.

Informed written consent for participation was obtained from each individual. The study was approved by the Korea Centers for Disease Control and Prevention Institutional Review Board.

### The methods of study and definition

#### ***1) Sociodemographic characteristics, occupational characteristics, and health behaviors***

For the sociodemographic variables, specifically, the subjects’ age, marital status, and education background, the data from the 11th year (2008) was used. For marital status, those who answered number (2) (“I am married and have a spouse”) were included in the married group, and those who answered (1) (“not married”), (3) (“separated”), (4) (“divorced”), or (5) (“widowed”) were included in the unmarried group. Education background was divided into three categories: middle school or lower, high school graduate and some college, and university graduate and higher.

For occupational characteristic variables, the occupation, employment type, tenure, shift system, and job satisfaction data from the 11th year (2008) were used. Occupations were divided into non-manual and manual categories. Non-manual occupations included managers, professionals, technicians, and clerks, while manual occupations included service and sales workers, agricultural and fishery workers, craft and related trade workers, plant and machine operators and assemblers, and elementary occupations. For employment type, the categorization standards were based on the study by Kim et al. (2008) [22]. We categorized workers as precarious or non-precarious. Nonstandard workers (i.e., workers in temporary help agencies, workers provided by contract firms, home-based workers, on-call workers, and independent contractors), contingent workers, and part-time workers were defined as atypical and thus precarious workers. The category of precarious workers also included temporary and daily workers. For the tenure variable, the subjects’ answer to the question, “When did you start working here (workplace, company)?” was subtracted from 2008, which was the year of investigation. To measure job satisfaction, we used responses to the prompt “I am satisfied with the work (job) I do now.” Answers number (1) (“No”) and number (2) (“Not really”) were graded as low job satisfaction, while answer number (3) (“Somewhat”) was graded as average satisfaction. Answers number (4) (“Mostly”) and (5) (“Very much”) were graded as high satisfaction.

For health behaviors, the question from the 11th year (2008) on drinking was used. The question, “Do you drink often?” was asked, and those who chose answer number (1) (“Yes”) were categorized as drinking, while those who chose number (2) (“I used to drink, but not anymore”) or number (3) (“I have never drunk”) were categorized as non-drinking.

#### ***2) Work hours***

In Korea, the legal work hours limit is up to 52 hours per week via article 50 and 53 of the Labor Standards Act. Whereas France and Germany set the legal upper limit of work hours as 35 hours per week in 1998 [23], due to bad economic conditions and decreasing profits, this law was modified to allow work hours up to 48 hours per week [24].

In this study, the points of reference for work-week length were 35 hours (the shortest regular work week in other countries) and 52 hours (the upper limit of weekly work hours in Korea). The subjects were divided into 3 groups for analysis, based on the data from the 11th year (2008): those who work less than 35 hours per week; those who work at least 35 hours but less than 52 hours per week; and those who work 52 hours or more per week. These groups were named the short work hours group, the reference work hours group, and the long work hours group, respectively.

Weekly work hours were defined as the sum of weekly regular work hours and overtime work hours. Weekly regular work hours were based on the answers to the question, “How long are your current weekly work hours, excluding meal times?” Weekly overtime work hours were based on the answers to the question, “What are your average overtime work hours per week?”.

#### ***3) Smoking***

In this study, smoking in 2008, smoking cessation, continuous smoking, and start of smoking were studied. Smoking prevalence was analyzed according to answers to the question, “Do you smoke?” Those answering (1) (“Yes”) were categorized as smokers. Smoking cessation was measured by the number of 2007 smokers who answered, in 2008, (2) (“I used to smoke but not anymore”) or (3) (“I don't smoke.”) Continuous smoking was defined as 2007 smokers who were still smoking in 2008, and start of smoking was measured by the number of 2008 smokers who were not smokers in 2007.

### Statistical methods

Direct standardization was used to calculate age-adjusted smoking, smoking cessation, continuous smoking, and start of smoking. Direct standardization calculates age-based prevalence by multiplying the number of people in each age group by the standard population, and then dividing the sum of the expected observation value by the total standard population. The standard population here consisted of males divided into age groups in five-year increments. The age-adjusted prevalence of smoking in the short work hours group, the reference work hours group, and the long work hours group, along with continuous smoking, smoking cessation, and start of smoking, were calculated with a 95% confidence interval.

Log-binomial regression analysis was used to study the relationship between weekly work hours and smoking behaviors in terms of the prevalence ratio (PR). Three models were constructed. In model 1, the baseline model, age was adjusted for to compare the smoking behaviors of the short work hours group, the reference work hours group, and the long work hours group. In model 2, age, education, and marital state were adjusted for. In model 3, age, education, marital state, drinking, and occupational characteristics, specifically, occupation, employment type, tenure, shifts, and job satisfaction, were adjusted for.

Relative explanatory power was used to assess the contribution of each explanatory factor to differences in smoking behaviors among the three groups. The relative explanatory power is defined by excessive risk decrease as a percentage when the explanatory variables vanish into the baseline model or previous model. The equation is [(PR in the baseline model)-(PR in the model adjusted for explanatory variables)]/[PR in the baseline model)-1]*100 [25]. If the PR in the previous model was 1.00, the relative explanatory power was not calculated. The SAS 9.1.2 package was used for analysis. All of the reported p values are two-tailed, and p<0.05 was considered to be significant.

## Results

### General characteristics of study subjects

Among the subjects, 4.9% of the workers fell into the short work hours group, 59.2% into the reference work hours group, and 36.0% into the long work hours group. Subjects aged ≥45 comprised 36.5% of the reference group. In the short work hours group, 46.0% of the subjects had a junior high school education or less, but most of the subjects in the reference and long work hours groups had at least a high-school education. There were significant differences in occupation and employment type according to work hours. In the short work hours group, 78.0% of the subjects were manual workers and 84.0% were precarious workers; in the reference group, 55.1% were non-manual workers and 79.0% were non-precarious workers; and in the long work hours group, 66.3% were manual workers and 82.3% were non-precarious workers. Tenure was 7.1±8.6 years in the short work hours group, 8.7±8.1 years in the reference work hours group, and 6.4±6.4 years in the long work hours group. Significant differences were also found in work shifts and job satisfaction. In the short work hours group, only 1.0% of the subjects were shift workers, compared to 8.9% of the reference work hours group and 20.5% of the long work hours group. In the short work hours group, 35% reported that their jobs were unsatisfying, compared to 8.4% of the reference group. Smoking varied according to work hours as well. In 2008, there were many smokers in all three groups (short work hours group 66.0%, reference work hours group 55.4%, and long work hours group 61.4%) (Table 1).

**Table 1:** **General characteristics of the study subjects N** (%)

**Variables**	**Weekly work hours**			**Total**	**p-value**
	Short*	**Reference†**	**Long‡**		
Total	100(4.9)	1209(59.2)	735(36.0)	2044	
Age (years)					
25-34	7(7.0)	353(29.2)	220(29.9)	580	<.0001
35-44	24(24.0)	414(34.2)	259(35.2)	697	
45-54	39(39.0)	305(25.2)	173(23.5)	517	
55-64	30(30.0)	137(11.3)	83(11.3)	250	
Marital status					
Married	68(68.0)	934(77.3)	545(74.2)	1547	0.056
Unmarried	32(32.0)	275(22.8)	190(25.9)	497	
Education					
≤Junior high school	46(46.0)	122(10.2)	110(15.0)	278	<.0001
High school	27(27.0)	372(30.8)	305(41.5)	704	
≥College	27(27.0)	715(59.1)	320(43.5)	1062	
Occupation					
Non-manual	22(22.0)	666(55.1)	248(33.7)	936	<.0001
Manual	78(78.0)	543(44.9)	487(66.3)	1108	
Employment type					
Precarious	84(84.0)	254(21.0)	130(17.7)	468	<.0001
Non-precarious	16(16.0)	955(79.0)	605(82.3)	1576	
Tenure (years) (Mean±S.D)	7.1±8.6	8.7±8.1	6.4±6.4		<.0001
Shift work					
Yes	1(1.0)	108(8.9)	151(20.5)	260	<.0001
No	99(99.0)	1101(91.1)	584(79.5)	1784	
Job satisfaction					
Low	35(35.0)	102(8.4)	102(13.9)	239	<.0001
Average	44(44.0)	550(45.5)	376(51.2)	970	
High	21(21.0)	557(46.1)	257(35.0)	835	
Smoking in 2007					
Yes	65(65.0)	629(52.0)	441(60.0)	1135	0.001
No	35(35.0)	580(48.0)	294(40.0)	909	
Smoking in 2008					
Yes	66(66.0)	670(55.4)	451(61.4)	1187	0.019
No	34(34.0)	539(44.6)	284(38.6)	857	
Alcohol drinking					
Yes	87(87.0)	1023(84.6)	627(85.3)	1737	0.776
No	13(13.0)	186(15.4)	108(14.7)	307	

*Weekly work hours less than 35, †Weekly work hours 35 or more, less than 52, ‡Weekly work hours more than 52.

### Smoking prevalence according to explanatory variables

In 2008, 41.9% of the subjects were non-smokers and 58.1% were smokers. Those aged 25–34 had the highest smoking prevalence (61.2%), and the prevalence decreased with age. Unmarried subjects had a higher smoking prevalence (65.6%) compared to married subjects (55.7%). The least educated group had the highest smoking prevalence (66.2%), and the higher the education, the lower the smoking prevalence. The smoking prevalence was higher among manual workers (63.6%) than among non-manual workers (51.5%), and higher in the precarious employment group (64.5%) than in the non-precarious employment group (56.2%). Smokers had shorter work tenure (nonsmokers 8.5±8.1 years, smokers 7.2±7.3 years). Those with low job satisfaction had the highest smoking prevalence (67.8%), and the prevalence decreased as satisfaction increased. Among 2007 smokers, 87.6% still smoked in 2008, while 12.4% of them quit smoking in 2008. Drinkers had a significantly higher smoking prevalence (62.4%) than did non-drinkers (33.6%) (Table 2).

**Table 2:** **Smoker proportion in 2008 by general characteristic subgroup N **(%)

**Variables**	**Smoking in 2008**		**Total**	**p-value**
	**No**	**Yes**		
Total	857(41.9)	1187(58.1)	2044	
Age (years)				
25-34	225(38.8)	355(61.2)	580	0.0002
35-44	282(40.5)	415(59.5)	697	
45-54	213(41.2)	304(58.8)	517	
55-64	137(54.8)	113(45.2)	250	
Marital status				
Married	686(44.3)	861(55.7)	1547	<.0001
Unmarried	171(34.4)	326(65.6)	497	
Education				
≤Junior high school	94(33.8)	184(66.2)	278	<.0001
High school	256(36.4)	448(63.6)	704	
≥College	507(47.7)	555(52.3)	1062	
Occupation				
Non-manual	454(48.5)	482(51.5)	936	<.0001
Manual	403(36.4)	705(63.6)	1108	
Employment type				
Precarious	166(35.5)	302(64.5)	468	0.0013
Non-precarious	691(43.9)	885(56.2)	1576	
Tenure (years) (Mean±S.D)	8.5±8.1	7.2±7.3		<.0001
Shift work				
Yes	120(46.2)	140(53.9)	260	0.1393
No	737(41.3)	1047(58.7)	1784	
Job satisfaction				
Low	77(32.2)	162(67.8)	239	<.0001
Average	370(38.1)	600(61.9)	970	
High	410(49.1)	425(50.9)	835	
Smoking in 2007				
Yes	141(12.4)	994(87.6)	1135	<.0001
No	716(78.8)	193(21.2)	909	
Alcohol drinking				
Yes	653(37.6)	1084(62.4)	1737	<.0001
No	204(66.5)	103(33.6)	307	

### Age-adjusted prevalence of smoking behaviors

The age-adjusted prevalence of smoking in 2008 was 57.6%, continuous smoking was 87.2%, smoking cessation was 12.8%, and start of smoking was 21.3%. The age-adjusted smoking prevalence was 64.9% in the short work hours group (95% confidence interval of 45.1-84.7), which was the highest; it was 54.7% in the reference work hours group (95% confidence interval of 50.5-59.0), which was the lowest. The age-adjusted continuous smoking prevalence was 88.1% in the long work hours group (95% confidence interval of 78.7-97.6), which was the highest, and the prevalence decreased with shorter work hours. Smoking cessation was 16.9% in the short work hours group (95% confidence interval of 1.1-32.8) which was the highest, and the prevalence decreased with longer work hours. Start of smoking was 33.2% in the short work hours group (95% confidence interval of 9.1-57.2), which was the highest (Table 3).

**Table 3:** **Age**-**adjusted prevalence and 95**% **confidence interval for smoking habits**

		Short work hours*	**Reference work hours†**	**Long work hours‡**	**Total**
Smoking in 2008	Number of population in 2008	100	1209	735	2044
	Number of smokers in 2008 (%)	66(66.0)	670(55.4)	451(61.4)	1187 (58.1)
	Age-adjusted prevalence	64.9(45.1-84.7)	54.7(50.5-59.0)	60.6(54.9-66.4)	57.6(54.3-60.9)
Continuous smoking§	Number of smokers in 2007	65	629	441	1135
	Number continuously smoking (%)	56(86.2)	549(87.3)	389(88.2)	994 (87.6)
	Age-adjusted prevalence	83.1(53.2-100.0)	86.8(79.3-94.4)	88.1(78.7-97.6)	87.2(81.6-92.8)
Smoking cessation∥	Number of smokers in 2007	65	629	441	1135
	Number who quit smoking (%)	9(13.8)	80(12.7)	52(11.8)	141(12.4)
	Age-adjusted prevalence	16.9(1.1-32.8)	13.2 (10.2-16.2)	11.9 (8.4-15.3)	12.8 (10.6-15.0)
Start smoking¶	Number of nonsmokers in 2007	35	580	294	909
	Number who started smoking (%)	10(28.6)	121(20.9)	62(21.1)	193(21.2)
	Age-adjusted prevalence	33.2(9.1-57.2)	20.7(17.0-24.4)	20.8(15.5-26.2)	21.3(18.2-24.3)

*Weekly work hours less than 35, †Weekly work hours 35 or more, less than 52, ‡Weekly work hours more than 52, §Smokers in 2008/smokers in 2007, ∥Adults quitting smoking in 2008/smokers in 2007, ¶Smokers in 2008/non-smokers in 2007.

### Prevalence ratios of smoking behaviors

When age was adjusted, the smoking prevalence of the short work hours group was 1.39 times higher than that of the reference work hours group (95% confidence interval of 1.17-1.65). When demographic variables were also adjusted for, the smoking prevalence of the short work hours group was 1.20 times higher than that of the reference work hours group (95% confidence interval of (1.01-1.42). The prevalence ratio decreased 48% from 1.39 to 1.20 by 48.7%. When the demographic variables, occupational variables, and alcohol drinking were all adjusted for, the prevalence decreased even more, and the statistical significance was lost. The prevalence ratio decreased 45% from 1.20 to 1.11.

With regard to starting smoking, the short work hours group had a ratio of 1.53 in model 1 (95% confidence interval of 0.88-2.66), 1.37 in model 2 (95% confidence interval of 0.78-2.43), and 1.39 in model 3 (95% confidence interval of 0.77-2.53), but it was not significant.

**Figure 1:**
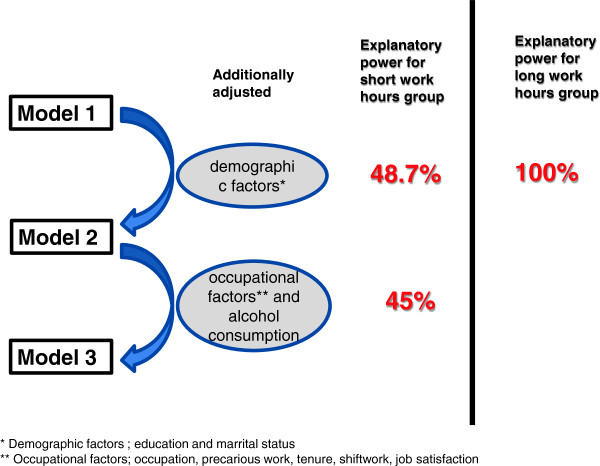
The relative explanatory power for smoking prevalence as a percentage reduction in the prevalence ratio.

Adjusted for age, the smoking prevalence of the long work hours group was 1.11 times higher than that of the reference work hours group (95% confidence interval of 1.03-1.19). When the demographic variables were also adjusted for, the smoking prevalence of the long work hours group was 1.00 times (95% confidence interval of 0.88-1.11) that of the reference group, and significance was lost (Table 4). This means that demographic variables alone explained the difference in smoking prevalence between the long work hours group and the reference group (Figure 1).

**Table 4:** **Prevalence ratio and 95**% **confidence interval for smoking habits**

	**Weekly work hours**	**Smoking in 2008**	**Continuous smoking§**	**Smoking cessation∥**	**Start smoking**¶
		**(n=2044)**	**(n=1135)**	**(n=1135)**	**(n=909)**
Model 1**	Short*	1.39(1.17-1.65)	1.00(0.90-1.10)	1.00(0.52-1.91)	1.53(0.88-2.66)
	Reference†	1	1	1	1
	Long‡	1.11(1.03-1.19)	1.01(0.96-1.06)	0.93(0.67-1.30)	1.02(0.78-1.34)
Model 2††	Short	1.20(1.01-1.42)	0.99(0.89-1.10)	1.06(0.55-2.05)	1.37(0.78-2.43)
	Reference	1	1	1	1
	Long	1.00(0.88-1.11)	1.00(0.96-1.05)	0.98(0.71-1.37)	0.96(0.73-1.27)
Model 3‡‡	Short	1.11(0.94-1.31)	0.97(0.88-1.08)	1.26(0.63-2.53)	1.36(0.75-2.47)
	Reference	1	1	1	1
	Long	0.95(0.84-1.06)	0.99(0.94-1.04)	1.06(0.75-1.48)	0.95(0.72-1.26)

*Weekly work hours more than 35, †Weekly work hours 35 or more, less than 52, ‡Weekly work hours more than 52, §Smokers in 2008/smokers in 2007, ∥Adults quitting smoking in 2008/smokers in 2007, ¶Smokers in 2008/non-smokers in 2007, **Model 1:adjusted for age, ††Model 2: adjusted for age, education, and marital status, ‡‡Model 3:adjusted for age, education, marital status, occupation, precarious work, tenure, shiftwork, job satisfaction, and alcohol consumption.

## Discussion

In this study, the short and long work hours groups had a higher smoking prevalence than the reference work hours group. Higher smoking prevalence among those who work long hours has also been reported in Spain, though the reference work hours were different from those in this study. Salaried female workers who work more than 40 hours per week had higher rates of smoking compared to those who worked 30–40 hours per week [16]. Furthermore, this is the first study, as far as we know, to report higher smoking prevalence among Koreans who work shorter hours. A previous study had divided work hours into two groups: a long work hours group and reference work hours group. Almost all studies defined a long work hours group as people who worked over 40 hours per week and a reference group as people who worked 40 hours per week or less [18,26-29]. In 2008, Lallukka presented a pooled analysis of the results of three previous prospective cohort studies. These cohort studies were the Whitehall II Study from London (n=3397), Helsinki Health Study (n=6070), and the Japanese Civil Servants Study (n=2213) [27]. There was no difference in the smoking prevalence between the long work hours group and short work hours group (reference group) in London and Helsinki, but in the Japanese study, the short work hours group had a statistically significantly higher odds ratio for smoking prevalence than the long work hours group. We believe that the difference in the smoking prevalence was what led to different results between Japan and London or Helsinki.

We investigated which factors explain the higher smoking prevalence in the short and long work hours groups. To answer this question, we explored the explanatory power of the demographic and occupational factors. The higher smoking prevalence in the short work hours group was explained by the subjects' education, marital status, and occupational characteristics. The higher smoking prevalence of the long work hours group was explained by the subjects' education and marital status. After adjusting for demographic and occupational factors, there were no direct or independent effects of weekly work hours. This means that short work hours and long work hours affect smoking behaviors through demographic or occupational characteristics, not directly.

All of the difference in smoking prevalence in the long work hours group compared to the reference group, and 48% of that in the short work hours group, were explained by demographic factors, specifically, education and marital status. Among the demographic variables, education was the biggest explanatory factor for the higher smoking prevalence in the long and short work hours groups. Actually, the average age of starting smoking in Korea was 19.3 for males [29], suggesting that most start smoking before getting a job. That means smoking characterized by nicotine dependency could be affected by the environment of one’s adolescence or young-adulthood. Education is used as the proxy for early-life environment. Education is also a strong determinant of smoking behaviors as well as occupation [30], which is, in turn, closely related to work hours. Many studies have found a relationship between low socioeconomic position—measured by education, income, or occupation—and high smoking prevalence [30-32]. Studies from other countries have also reported that smoking tends to begin in adolescence or early adulthood, and that it is strongly related to education level [31]. Therefore, the high smoking prevalence of the long and short work hours groups is probably determined before they begin their worklives, and this can be explained by their low socioeconomic position.

After 48% of the short work hours group’s higher smoking prevalence was explained by education and marital status, 45% of the remnant was explained by occupational characteristics. The short work hours group, reference work hours group, and long work hours group had significant differences in their occupational characteristics. The short work hours group had the highest proportion of manual workers and precarious workers, as well as the lowest job satisfaction. The highest rate of taking up smoking among the short work hours group may be related to these occupational characteristics aswell. Previous studies have reported a relationship between low job satisfaction and/or precarious employment [33] on the one hand, and smoking and nicotine dependency [34] on the other. One previous study reported that precarious workers has significantly higher job insecurity and psychosocial stress [35]. If psychosocial stress worsens, the risk for smoking increases [13]. Another study asked current smoker what their dominant motives were for smoking. The most common answer was 'habit', the second most common reason to smoke was 'work-related stress'(34.5%). That study tried to determine the causal pathway from job stress to smoking. The job stress had a statistically significant relationship with depression, and depression had a significant relationship with smoking. Thus, we were able to determine that job stress has a direct relationship with depression, and has an indirect relationship with smoking via depression [36]. Workers who engaged in precarious jobs were more likely to be under psychological stress and/or be smokers. In this study, the most important factors explaining high smoking prevalence were education, employment type, and job satisfaction. The results imply that poor employment type and the low job satisfaction of workers with short work hours could be a key point of intervention in reducing smoking prevalence.

The prevalence ratios of continuous smoking and smoking cessation were not significant when the short and long work hours groups were compared with the reference group in this study. This corresponds with research results in Denmark that reported no association between work hours and cessation rates in 3606 Danish workers [37]. However, a previous cohort study in Norway reported that longer weekly work hours lower the odds of smoking cessation in nurses’ aides [24]. What distinguishes the Norway study from this and the Danish study are the study subjects. The subjects in the Norway study had only one job title, and most of them were women. In contrast, the subjects in this study were men with various jobs. The difference in the degree of nicotine dependence in the study subjects could be connected to the success of smoking cessation. This points to the need for more detailed cohort studies on smoking behaviors.

This study has a few limitations. First, not enough members of the sample who had quit smoking were secured in the short work hours group. Secondly, the answer (1) (“Yes”) to the question, “Do you smoke?” provides no information as to the amount of smoking. Having an insufficient sample or no information about the extent could have minimized the degree of association. Thirdly, females were excluded. Female smoking is continuously rising in Korea, and there are differences between men and women [38] in the factors related to smoking. Further studies are needed to explore smoking behaviors in women.

However, the strengths of this study are as follows: First, data from KLIPS was used, and this data is strongly representative. Second, not only were the relationship between work hours, smoking prevalence, and smoking cessation analyzed, but continuous smoking and start of smoking were also considered in order to explore the behavior in depth. Third, previous studies merely analyzed the difference between a long work hours group and a control group [18,19,39]. However, this study divided the groups into short, reference, and long work hours groups, which should more accurately capture the reality of those who feel they are working too little, about average, or too much according to societal norms and thus distinguish between the norm and those disadvantaged by or frustrated with under- or overwork. Fourth, prevalence ratios were used to decrease the error of the odds ratio. In case the dependent variable has a high prevalence, the odds ratio is limited in estimating the relative risk.

To date, studies in Korea on long work hours and smoking behaviors are scarce, and this study can be viewed as a contribution to research on the work environment and its relationship with smoking in Korea. In order to decrease the smoking prevalence in the short work hours group, job-related factors like job satisfaction and employment type need to be considered. More research is needed to determine whether long work hours are related to continuous smoking and smoking cessation.

## Conclusion

Any intervention program to decrease the smoking prevalence in those with short work hours must take into account employment type, job satisfaction, and work-related factors.

## Competing interests

The authors declare that they have no competing interests.

## Authors’ contributions

K Jung-Choi conceived of and designed the study. All of the authors developed the research model, and S-M Jang analyzed the statistics and wrote the manuscript. All of the authors read and approved the final manuscript.
